# Supplementary material for: In Search of Apis mellifera pomonella in Kazakhstan
Source: Life (Basel). 2023 Sep 3;13(9):1860. doi: 10.3390/life13091860 (PMC10532534; doi:10.3390/life13091860)
Supplement: Supplementary file 1 [file life-13-01860-s001.zip › Supplementary-document-S1.html]

In search of Apis mellifera pomonella in Kazakhstan


# In search of *Apis mellifera pomonella* in Kazakhstan

#### Kamshat Temirbayeva, Aibyn Torekhanov, Ulzhan Nuralieva, Zhanar Sheralieva, Adam Tofilski

#### 2023-08-18

#### Affiliations

Kamshat Temirbayeva
Kazakh Research Institute of Livestock and Fodder Production, al-Farabi
Kazakh National University, Kazakhstan

Aibyn Torekhanov
Kazakh Research Institute of Livestock and Fodder Production,
Kazakhstan

Ulzhan Nuralieva
Kazakh Research Institute of Livestock and Fodder Production,
Kazakhstan

Zhanar Sheralieva
Kazakh Research Institute of Livestock and Fodder Production,
Kazakhstan

Adam Tofilski
University of Agriculture in Krakow, Poland

### Libraries

```
# calculations
library(geomorph) # GPA 
library(shapes) # OPA
library(Morpho) # CVA
library(MASS) # LDA
# plotting and visualization
library(ggplot2) # plots
ggplot2::theme_set(theme_light())
library(ggforce) # geom_ellipse
library(ggrepel) # geom_label_repel
library(rnaturalearth) # maps
library(raster) # raster
library(ggspatial) # annotation_scale
options(digits=4) # number of digits to display
```

### Variable names

In order to avoid mistakes it is safer to use columns names instead
of indexes

```
p <- 19  # number of landmarks
k <- 2  # number of dimensions, in this case 2 for coordinates (x, y)

# create coordinates names used by IdentiFly
xyNames <- c("x1", "y1")
for (i in 2:p) {
  xyNames <- c(xyNames, paste0("x", i))
  xyNames <- c(xyNames, paste0("y", i))
}
xyNames
```

```
##  [1] "x1"  "y1"  "x2"  "y2"  "x3"  "y3"  "x4"  "y4"  "x5"  "y5"  "x6"  "y6" 
## [13] "x7"  "y7"  "x8"  "y8"  "x9"  "y9"  "x10" "y10" "x11" "y11" "x12" "y12"
## [25] "x13" "y13" "x14" "y14" "x15" "y15" "x16" "y16" "x17" "y17" "x18" "y18"
## [37] "x19" "y19"
```

```
# create coordinates names used by geomorph
xy.Names <- c("1.X", "1.Y")
for (i in 2:p) {
  xy.Names <- c(xy.Names, paste(i, "X", sep = "."))
  xy.Names <- c(xy.Names, paste(i, "Y", sep = "."))
}
xy.Names
```

```
##  [1] "1.X"  "1.Y"  "2.X"  "2.Y"  "3.X"  "3.Y"  "4.X"  "4.Y"  "5.X"  "5.Y" 
## [11] "6.X"  "6.Y"  "7.X"  "7.Y"  "8.X"  "8.Y"  "9.X"  "9.Y"  "10.X" "10.Y"
## [21] "11.X" "11.Y" "12.X" "12.Y" "13.X" "13.Y" "14.X" "14.Y" "15.X" "15.Y"
## [31] "16.X" "16.Y" "17.X" "17.Y" "18.X" "18.Y" "19.X" "19.Y"
```

```
# The number of principal components used is 2*p-4 = 34, which is equal to the
# degrees of freedom create principal components names used by prcomp
pcNames <- paste0("PC", 1:(2 * p - 4))
pcNames
```

```
##  [1] "PC1"  "PC2"  "PC3"  "PC4"  "PC5"  "PC6"  "PC7"  "PC8"  "PC9"  "PC10"
## [11] "PC11" "PC12" "PC13" "PC14" "PC15" "PC16" "PC17" "PC18" "PC19" "PC20"
## [21] "PC21" "PC22" "PC23" "PC24" "PC25" "PC26" "PC27" "PC28" "PC29" "PC30"
## [31] "PC31" "PC32" "PC33" "PC34"
```

```
geoNames <- c("latitude", "longitude")
```

### Read landmark coordinates

```
wings <- read.csv("https://zenodo.org/record/8128010/files/KZ-raw-coordinates.csv")

# extract sample classifier
wings$sample = substr(wings$file,1,7)
head(wings, 2)
```

```
##                         file  x1  y1  x2  y2  x3  y3  x4  y4  x5  y5  x6  y6
## 1 KZ-0001-000001_c10C.dw.png 264 274 289 271 352 375 351 301 366 190 449 378
## 2 KZ-0001-000002_c11C.dw.png 347 260 380 259 432 369 438 292 449 174 531 376
##    x7  y7  x8  y8  x9  y9 x10 y10 x11 y11 x12 y12 x13 y13 x14 y14 x15 y15 x16
## 1 520 416 496 387 553 339 505 314 562 267 573 216 590 170 609 401 651 349 751
## 2 607 414 583 388 636 339 599 314 653 263 666 213 685 166 699 402 754 349 849
##   y16 x17 y17 x18 y18 x19 y19  sample
## 1 298 806 286 818 245 100 379 KZ-0001
## 2 298 894 290 909 247 189 363 KZ-0001
```

### Map of sampling locations

```
# Read geographic coordinates
geo.data <- read.csv("https://zenodo.org/record/8128010/files/KZ-data.csv", strip.white=TRUE)
geo.data.sample <- aggregate(geo.data[geoNames], by = list(geo.data$sample), FUN = mean)
rownames(geo.data.sample) = geo.data.sample$Group.1
geo.data.sample <- subset(geo.data.sample, select = -c(Group.1))

sample.gr = geo.data[,c("sample", "group")]
sample.gr = sample.gr[!duplicated(sample.gr), ]
geo.data.sample$group = sample.gr$group
table(geo.data.sample$group)
```

```
## 
##   carnica local bee mellifera 
##        38        29         4
```

```
locations = geo.data[,geoNames]
locations = locations[!duplicated(locations), ]

# Read elevation data
# In order to download the TIF file uncomment the two lines below
# download.file("https://geodata.ucdavis.edu/climate/worldclim/2_1/base/wc2.1_2.5m_elev.zip", "wc2.1_2.5m_elev.zip")
# unzip("wc2.1_2.5m_elev.zip")
elev <- raster("E:/WorldClim/wc2.1_2.5m_elev.tif")

elev.color <- colorRampPalette(c("#f7f7f7", "#f0f0f0", "#d9d9d9", "#bdbdbd", "#636363"))

x.min = 45
x.max = 90
y.min = 35
y.max = 56
e <- extent(x.min, x.max, y.min, y.max)
elev = crop(elev, e)
elev.df  <-  data.frame(rasterToPoints(elev))
colnames(elev.df) = c("longitude", "latitude", "altitude")

world <- ne_countries(scale = "medium", returnclass = "sf")

# Chen et al. 2016 locations
Chen = data.frame(latitude = c(43.2941, 43.3019,    43.2975,    43.3013),
                  longitude = c(83.5897,    83.6633,    83.7133,    83.6663), 
                  group = "Chen et al. 2016")

# Sheppard and Meixner 2003 locations
Sheppard = data.frame(latitude = c(42.3527, 43.0203,    43.1666))
Sheppard$longitude = c(70.3775, 78.2894,    79.2943)
Sheppard$group = "Shepard and Meixner 2003"

map.coord = rbind(geo.data.sample, Chen, Sheppard)
# jitter the coordinates to show all locations
jitter.x <- jitter(map.coord$longitude, amount = 0.2)
jitter.y <- jitter(map.coord$latitude, amount = 0.2)

ggplot(data = world) +
  geom_sf() +
  coord_sf(xlim = c(30, 130), ylim = c(10, 60)) +
  annotate("rect", xmin = x.min, xmax = x.max, ymin = y.min, ymax = y.max, alpha = 0, color= "red")
```

```
ggplot(data = world) +
  geom_sf() +
  geom_point(data = map.coord, aes(x = jitter.x, y = jitter.y, 
                                         colour = group, stroke = 1), size = 1) +
  scale_color_manual(name ="", 
                     values = c("red", "blue", "green", "black", "magenta"),
                     breaks=c('local bee', 'carnica', 'mellifera', 
                               'Shepard and Meixner 2003','Chen et al. 2016'),
                      labels=c('local bees', 'A. m. carnica', 'A. m. mellifera', 
                               'Shepard and Meixner 2003','Chen et al. 2016')) +
  coord_sf(xlim = c(x.min, x.max), ylim = c(y.min, y.max), expand = FALSE) +
  theme(axis.title.x=element_blank(), axis.title.y=element_blank())
```

```
ggplot(data = world) +
  geom_raster(data = elev.df, aes(longitude, latitude, fill = altitude)) +
  scale_fill_gradientn(colours = elev.color(100)) +
  geom_sf(fill = NA) +
  geom_point(data = map.coord, aes(x = jitter.x, y = jitter.y, 
                                         colour = group, stroke = 1), size = 1) +
  scale_color_manual(name ="", 
                     values = c("red", "blue", "green", "black", "magenta"),
                     breaks=c('local bee', 'carnica', 'mellifera', 
                               'Shepard and Meixner 2003','Chen et al. 2016'),
                      labels=c('local bees', 'A. m. carnica', 'A. m. mellifera', 
                               'Shepard and Meixner 2003','Chen et al. 2016')) +
  coord_sf(xlim = c(67, 84), ylim = c(38, 47)) +
  theme(axis.title.x=element_blank(), axis.title.y=element_blank()) + 
  annotation_scale(location = "tl", width_hint = 0.2)
```

### GPA-alignment

```
# Convert from 2D array to 3D array
wings.coords <- arrayspecs(wings[xyNames], p, k)
dimnames(wings.coords)[[3]] <- wings$file

# Align the coordinates using Generalized Procrustes Analysis 
GPA <- gpagen(wings.coords, print.progress = FALSE)
consensus = GPA$consensus

# # plot landmarks after alignment
# plotAllSpecimens(GPA$coords,links=links.apis,label=TRUE,
#                  plot.param = list(pt.bg = "black", pt.cex = 0.5, 
#                                    mean.bg = "red", mean.cex=1, link.col="red", 
#                                    txt.pos=3, txt.cex=1))

# Convert from 3D array to 2D array
wings.aligned <- two.d.array(GPA$coords) 
colnames(wings.aligned) = xyNames
head(wings.aligned, 2)
```

```
##                                 x1       y1      x2       y2      x3      y3
## KZ-0001-000001_c10C.dw.png -0.2793 -0.05838 -0.2499 -0.05997 -0.1843 0.06623
## KZ-0001-000002_c11C.dw.png -0.2831 -0.06161 -0.2451 -0.06130 -0.1901 0.06756
##                                 x4       y4      x5      y5       x6      y6
## KZ-0001-000001_c10C.dw.png -0.1798 -0.02022 -0.1538 -0.1486 -0.07131 0.07714
## KZ-0001-000002_c11C.dw.png -0.1798 -0.02076 -0.1619 -0.1560 -0.07652 0.08001
##                                  x7     y7       x8      y8      x9      y9
## KZ-0001-000001_c10C.dw.png 0.008661 0.1269 -0.01714 0.09124 0.05306 0.03956
## KZ-0001-000002_c11C.dw.png 0.009229 0.1271 -0.01723 0.09612 0.04592 0.04210
##                                  x10      y10     x11      y11     x12     y12
## KZ-0001-000001_c10C.dw.png -0.001055 0.006718 0.06906 -0.04379 0.08580 -0.1025
## KZ-0001-000002_c11C.dw.png  0.004462 0.011693 0.06885 -0.04458 0.08602 -0.1015
##                               x13     y13    x14    y14    x15     y15    x16
## KZ-0001-000001_c10C.dw.png 0.1092 -0.1549 0.1137 0.1162 0.1667 0.05872 0.2873
## KZ-0001-000002_c11C.dw.png 0.1100 -0.1548 0.1156 0.1174 0.1812 0.05884 0.2928
##                                 y16    x17       y17    x18      y18     x19
## KZ-0001-000001_c10C.dw.png 0.006832 0.3524 -0.002972 0.3696 -0.04991 -0.4787
## KZ-0001-000002_c11C.dw.png 0.004376 0.3449 -0.002831 0.3641 -0.05164 -0.4694
##                                y19
## KZ-0001-000001_c10C.dw.png 0.05165
## KZ-0001-000002_c11C.dw.png 0.04988
```

### Average aligned coordinates within samples

```
sample.aligned <- aggregate(wings.aligned, by = list(wings$sample), FUN = mean)
rownames(sample.aligned) = sample.aligned$Group.1
sample.aligned <- subset(sample.aligned, select = -c(Group.1))

sample.aligned$group = sample.gr$group

head(sample.aligned, 2)
```

```
##              x1       y1      x2       y2      x3      y3      x4       y4
## KZ-0001 -0.2817 -0.06049 -0.2501 -0.06247 -0.1805 0.06655 -0.1777 -0.01958
## KZ-0002 -0.2815 -0.05963 -0.2476 -0.06147 -0.1844 0.06520 -0.1784 -0.01907
##              x5      y5       x6      y6       x7     y7       x8      y8
## KZ-0001 -0.1641 -0.1508 -0.06890 0.07734 0.009142 0.1278 -0.01484 0.09266
## KZ-0002 -0.1638 -0.1471 -0.06891 0.07552 0.012188 0.1267 -0.01176 0.09120
##              x9      y9      x10      y10     x11      y11     x12     y12
## KZ-0001 0.04752 0.04263 0.004954 0.009638 0.07003 -0.04236 0.08267 -0.1033
## KZ-0002 0.05041 0.04329 0.005981 0.009975 0.06791 -0.04037 0.07971 -0.1004
##            x13     y13    x14    y14    x15     y15    x16      y16    x17
## KZ-0001 0.1045 -0.1567 0.1136 0.1180 0.1723 0.06210 0.2945 0.003492 0.3493
## KZ-0002 0.1010 -0.1552 0.1138 0.1174 0.1737 0.06184 0.2974 0.002025 0.3493
##               y17    x18      y18     x19     y19   group
## KZ-0001 -0.002553 0.3644 -0.04886 -0.4750 0.04691 carnica
## KZ-0002 -0.002603 0.3636 -0.04937 -0.4786 0.04216 carnica
```

### Principal component analysis of the samples

```
sample.pca <- prcomp(sample.aligned[ , xyNames])
sample.pca.scores <- as.data.frame(sample.pca$x)
sample.pca.scores$group = sample.aligned$group

# create plot labels
variance.tab <- summary(sample.pca)$importance
variance <- variance.tab["Proportion of Variance", "PC1"]
variance <- round(100 * variance, 1)
label.x <- paste0("PC1 (", variance, "%)")
variance <- variance.tab["Proportion of Variance", "PC2"]
variance <- round(100 * variance, 1)
label.y <- paste0("PC2 (", variance, "%)")

ggplot(sample.pca.scores, aes(x = PC1, y = PC2, shape = group, color = group)) +
  geom_point() +
  scale_shape_manual(name ="", values = c(15:17),
                     breaks=c('local bee', 'carnica', 'mellifera'),
                     labels=c('local bees', 'A. m. carnica', 'A. m. mellifera')) +
  scale_color_manual(name ="", 
                     values = c("red", "blue", "green"),
                     breaks=c('local bee', 'carnica', 'mellifera'),
                     labels=c('local bees', 'A. m. carnica', 'A. m. mellifera')) +
  stat_ellipse() + 
  xlab(label.x) + ylab(label.y)
```

### MANOVA

```
MANOVA.samples = manova(as.matrix(cbind(sample.pca.scores[, pcNames])) ~ group, sample.pca.scores)
summary(MANOVA.samples)
```

```
##           Df Pillai approx F num Df den Df Pr(>F)    
## group      2   1.89       19     68     72 <2e-16 ***
## Residuals 68                                         
## ---
## Signif. codes:  0 '***' 0.001 '**' 0.01 '*' 0.05 '.' 0.1 ' ' 1
```

### Canonical variate analysis

```
# use equal prior probability for all groups
n.country <- length(unique(sample.pca.scores$group)) # number of groups
sample.cva <- CVA(sample.pca.scores[pcNames], sample.pca.scores$group, 
                  rounds = 10000, cv = TRUE,
                  prior = rep(1/n.country, n.country))
sample.cva.scores <- as.data.frame(sample.cva$CVscores)
# remove unwanted spaces in variable names otherwise use `CV 1`
colnames(sample.cva.scores) <- gsub(" ", "", colnames(sample.cva.scores)) 
# sample.cva.scores <- cbind(sample.geo.data, sample.cva.scores)
sample.cva.scores$group = sample.aligned$group

ggplot(sample.cva.scores, aes(x = CV1, y = CV2, shape = group, color = group)) +
  geom_point() +
  scale_shape_manual(name ="", values = c(15:17),
                     breaks=c('local bee', 'carnica', 'mellifera'),
                     labels=c('local bees', 'A. m. carnica', 'A. m. mellifera')) +
  scale_color_manual(name ="", 
                     values = c("red", "blue", "green"),
                     breaks=c('local bee', 'carnica', 'mellifera'),
                     labels=c('local bees', 'A. m. carnica', 'A. m. mellifera')) +
  stat_ellipse()
```

```
# Confusion matrix
# Classification of the samples to groups
CVA.class <- typprobClass(sample.cva$CVscores, groups = as.factor(sample.cva.scores$group), outlier = 0)
print(CVA.class)
```

```
##  cross-validated classification results in frequencies
##            
##             carnica local bee mellifera
##   carnica        38         0         0
##   local bee       0        29         0
##   mellifera       0         0         4
## 
## 
##  cross-validated classification result in %
##            
##             carnica local bee mellifera
##   carnica       100         0         0
##   local bee       0       100         0
##   mellifera       0         0       100
## 
## 
##  overall classification accuracy: 100 %
## 
##  Kappa statistic: 1
```

```
# Mahalanobis distances between groups
knitr::kable(as.data.frame(as.matrix(sample.cva$Dist$GroupdistMaha)), digits = 3)
```

|  | carnica | local bee | mellifera |
| --- | --- | --- | --- |
| carnica | 0.000 | 9.269 | 17.64 |
| local bee | 9.269 | 0.000 | 17.39 |
| mellifera | 17.643 | 17.385 | 0.00 |

```
# Significance of differences between groups
knitr::kable(as.data.frame(as.matrix(sample.cva$Dist$probsMaha)))
```

|  | carnica | local bee | mellifera |
| --- | --- | --- | --- |
| carnica | 0e+00 | 1e-04 | 1e-04 |
| local bee | 1e-04 | 0e+00 | 1e-04 |
| mellifera | 1e-04 | 1e-04 | 0e+00 |

### Cubital index

```
a = ((sample.aligned$x1-sample.aligned$x2)^2+
                      (sample.aligned$y1-sample.aligned$y2)^2)^0.5
cubital = data.frame(a = a)
cubital$b = ((sample.aligned$x2-sample.aligned$x4)^2+
                      (sample.aligned$y2-sample.aligned$y4)^2)^0.5
cubital$ci = cubital$b/cubital$a
cubital$group = sample.aligned$group

aggregate(cubital$ci, by = list(cubital$group), FUN=mean)
```

```
##     Group.1     x
## 1   carnica 2.693
## 2 local bee 2.305
## 3 mellifera 1.720
```

```
aggregate(cubital$ci, by = list(cubital$group), FUN=sd)
```

```
##     Group.1      x
## 1   carnica 0.2174
## 2 local bee 0.2235
## 3 mellifera 0.1246
```

```
ANOVA = aov(ci ~ group, data = cubital)
summary(ANOVA)
```

```
##             Df Sum Sq Mean Sq F value  Pr(>F)    
## group        2   4.91   2.457    52.3 1.8e-14 ***
## Residuals   68   3.19   0.047                    
## ---
## Signif. codes:  0 '***' 0.001 '**' 0.01 '*' 0.05 '.' 0.1 ' ' 1
```

### Comparison with honey bee lineages

Read landmark coordinates from lineages dataset
Nawrocka et al., (2018a) and
Nawrocka et al., (2018b)

```
wings.lin <- read.csv("https://zenodo.org/record/7567336/files/Nawrocka_et_al2018.csv", header = TRUE)

# extract sample classifier
wings.lin$sample = substr(wings.lin$file,1,10)
wings.lin$lineage = substr(wings.lin$file,1,1)
wings.lin$subspecies = substr(wings.lin$file,1,5)
head(wings.lin, 2)
```

```
##                   file  x1  y1  x2  y2  x3  y3  x4  y4  x5  y5  x6  y6  x7  y7
## 1 A-ada-0698-01.dw.png 191 229 207 227 239 276 239 240 238 185 295 272 331 288
## 2 A-ada-0698-02.dw.png 166 243 182 241 212 295 216 258 218 204 270 294 303 312
##    x8  y8  x9  y9 x10 y10 x11 y11 x12 y12 x13 y13 x14 y14 x15 y15 x16 y16 x17
## 1 319 274 341 249 325 240 344 214 342 188 348 165 372 276 392 249 438 219 460
## 2 291 297 318 274 301 263 323 240 326 214 336 192 346 302 369 276 414 249 441
##   y17 x18 y18 x19 y19     sample lineage subspecies
## 1 214 461 191 118 289 A-ada-0698       A      A-ada
## 2 245 446 224  91 295 A-ada-0698       A      A-ada
```

### GPA-alignment of lineages dataset

Before analysis all landmark configurations have to be
superimposed.

```
# Convert 2D array into a 3D array
wings.lin.3D <- arrayspecs(wings.lin[xyNames], p, k)
dimnames(wings.lin.3D)[[3]] <- wings.lin$file
# Align the coordinates using Generalized Procrustes Analysis 
GPA.lin <- gpagen(wings.lin.3D, print.progress = FALSE)
# Convert 3D array into a 2D array - opposite to arrayspecs
wings.lin.aligned <- two.d.array(GPA.lin$coords)
```

### Average aligned coordinates within samples for lineages dataset

```
sample.lin.aligned <- aggregate(wings.lin.aligned, by = list(wings.lin$sample), FUN = mean)
names(sample.lin.aligned)[names(sample.lin.aligned) == "Group.1"] <- "sample" # rename column

# extract lineage code as classifier
sample.lin.aligned$lineage = substr(sample.lin.aligned$sample,1,1)

geo.data.lin <- read.csv("https://zenodo.org/record/7567336/files/Nawrocka_et_al2018-geo-data.csv", header = TRUE)
```

### Canonical variate analysis based on lineages

```
# Convert 2D array into a 3D array for easier analysis of unknown samples
sample.lin.3D <- arrayspecs(sample.lin.aligned[xy.Names], p, k)
dimnames(sample.lin.3D)[[3]] <- sample.lin.aligned$sample

# use equal prior probability for all groups
n.lin <- length(unique(sample.lin.aligned$lineage)) # number of groups
sample.lin.cva <- CVA(sample.lin.3D, sample.lin.aligned$lineage, rounds = 10000, cv = TRUE, 
                      prior = rep(1/n.lin, n.lin))
```

```
## singular Covariance matrix: General inverse is used. Threshold for zero eigenvalue is 1e-10
```

```
sample.lin.cva.scores <- as.data.frame(sample.lin.cva$CVscores)
# remove unwanted spaces in variable names otherwise use `CV 1`
colnames(sample.lin.cva.scores) <- gsub(" ", "", colnames(sample.lin.cva.scores)) 

sample.lin.cva.scores <- cbind(sample.lin.aligned$lineage, sample.lin.cva.scores)
names(sample.lin.cva.scores)[1] <- "lineage" # rename column
rownames(sample.lin.cva.scores) <- sample.lin.aligned$sample

ggplot(sample.lin.cva.scores, aes(x = CV1, y = CV2, color = lineage)) +
  geom_point() +
  coord_fixed() +
  stat_ellipse()
```

### Classification of the samples to lineages

```
# Convert 2D array into a 3D array
sample.only <- sample.aligned[xyNames]
unknown.wings <- arrayspecs(sample.only, p, k)
dimnames(unknown.wings)[[3]] <- sample.only$sample

# calculate covarience for each lineage
covariances <- lapply(unique(sample.lin.cva.scores$lineage),
                      function(x)
                        cov(sample.lin.cva.scores[sample.lin.cva.scores$lineage==x,-1]))
means <- aggregate(sample.lin.cva.scores[,names(sample.lin.cva.scores) != "lineage"], 
                  list(sample.lin.cva.scores$lineage), FUN=mean)
rownames(means) <- means$Group.1
means <- means[,names(means) != "Group.1"]

# Projection of the samples to canonical variate space from Nawrocka et al. 2018
groups <- rownames(means)
result.list <- vector(mode = "list", length = nrow(sample.aligned))
CV.list <- vector(mode = "list", length = nrow(sample.aligned))
for (r in 1:nrow(sample.aligned)) { 
  # Align unknown consensus with consensus from reference samples
  unknown.OPA <- procOPA(GPA.lin$consensus, unknown.wings[,,r])
  unknown.aligned <- unknown.OPA$Bhat
  CV.row <- predict(sample.lin.cva, unknown.aligned)

  # create empty list for results
  result <- numeric(length(groups))
  for (i in 1:length(groups)) { 
    MD <- mahalanobis(CV.row, unlist(means[i, ]), as.matrix(covariances[[i]]))
    result[i] <- MD
  }
  result.list[[r]] <- result
  CV.list[[r]] <- CV.row
}
CV.tab <- do.call(rbind, CV.list)
# remove unwanted spaces in variable names otherwise use `CV 1`
colnames(CV.tab) <- gsub(" ", "", colnames(CV.tab)) 
CV.tab <- as.data.frame(CV.tab)
CV.tab$group <- sample.aligned$group
rownames(CV.tab) = rownames(sample.aligned)

# Black ellipses A, C, M and O indicate 95% confidence regions of reference samples from Nawrocka et al. 2018
ggplot(CV.tab, aes(x = CV1, y = CV2, shape = group, color = group))+
  geom_point() +
  scale_shape_manual(name ="", values = c(15:17),
                     breaks=c('local bee', 'carnica', 'mellifera'),
                     labels=c('local bees', 'A. m. carnica', 'A. m. mellifera')) +
  scale_color_manual(name ="", 
                     values = c("red", "blue", "green"),
                     breaks=c('local bee', 'carnica', 'mellifera'),
                     labels=c('local bees', 'A. m. carnica', 'A. m. mellifera')) +
  stat_ellipse() +
  stat_ellipse(data = subset(sample.lin.cva.scores, lineage == "A"), 
               mapping = aes(x = CV1, y = CV2), inherit.aes = FALSE) + 
  stat_ellipse(data = subset(sample.lin.cva.scores, lineage == "C"), 
               mapping = aes(x = CV1, y = CV2), inherit.aes = FALSE) + 
  stat_ellipse(data = subset(sample.lin.cva.scores, lineage == "M"), 
               mapping = aes(x = CV1, y = CV2), inherit.aes = FALSE) + 
  stat_ellipse(data = subset(sample.lin.cva.scores, lineage == "O"), 
               mapping = aes(x = CV1, y = CV2), inherit.aes = FALSE) + 
  geom_label(means, 
             mapping = aes(x = CV1, y = CV2, label = rownames(means)), inherit.aes = FALSE)
```

```
MD.tab <- do.call(rbind, result.list)
MD.tab <- sqrt(MD.tab)
# column names for lineages
colnames(MD.tab) <- groups
MD.tab <- as.data.frame(MD.tab)

lineage <- colnames(MD.tab)[apply(MD.tab, 1, which.min)]
# final column names
colnames(MD.tab) <- paste0("MD.",groups)
MD.tab <- cbind(MD.tab, lineage)
rownames(MD.tab) <- sample.aligned$sample

# frequencies of the lineages
table(MD.tab$lineage)
```

```
## 
##  A  C  M  O 
##  8 49  2 12
```

```
# frequencies of the lineage in countries
table(sample.aligned$group, MD.tab$lineage)
```

```
##            
##              A  C  M  O
##   carnica    0 38  0  0
##   local bee  6 11  0 12
##   mellifera  2  0  2  0
```

```
head(CVA.class$probs)
```

```
##         carnica local bee mellifera
## carnica  0.4901 3.708e-19 4.896e-77
## carnica  0.8432 3.796e-20 1.104e-64
## carnica  0.6932 1.122e-16 2.099e-63
## carnica  0.4775 2.003e-17 1.720e-59
## carnica  0.2197 9.410e-14 3.820e-71
## carnica  0.9316 6.330e-21 5.022e-69
```

```
head(CVA.class$groupaffin)
```

```
## [1] carnica carnica carnica carnica carnica carnica
## Levels: carnica local bee mellifera
```

```
head(CVA.class$probsCV)
```

```
##         carnica local bee mellifera
## carnica  0.4689 6.919e-19 2.428e-77
## carnica  0.8368 7.125e-20 5.819e-64
## carnica  0.6803 1.487e-16 7.109e-63
## carnica  0.4558 3.165e-17 6.780e-60
## carnica  0.1920 5.268e-14 2.511e-70
## carnica  0.9288 1.137e-20 4.966e-68
```

```
p.local = CVA.class$probs
colnames(p.local)[2] = "pomonella"
p.local = subset(p.local, )
```

### Discrimination between local bees and evilutionary lineages

```
# use only local bees
KZ.lin = subset(sample.aligned, group == "local bee")

# format lineage data
lin.tmp = sample.lin.aligned[, xy.Names]
rownames(lin.tmp) = sample.lin.aligned$sample
colnames(lin.tmp) = xyNames
lin.tmp$group = sample.lin.aligned$lineage

KZ.lin = rbind(KZ.lin, lin.tmp)
head(KZ.lin, 2)
```

```
##              x1       y1      x2       y2      x3      y3      x4       y4
## KZ-0041 -0.2784 -0.05707 -0.2361 -0.05729 -0.1949 0.06576 -0.1752 -0.01745
## KZ-0042 -0.2754 -0.05717 -0.2422 -0.05842 -0.1909 0.06448 -0.1810 -0.02003
##              x5      y5       x6      y6       x7     y7       x8      y8
## KZ-0041 -0.1563 -0.1487 -0.06587 0.07741 0.010014 0.1285 -0.01531 0.09147
## KZ-0042 -0.1611 -0.1465 -0.06797 0.07672 0.008286 0.1247 -0.01707 0.09101
##              x9      y9      x10      y10     x11      y11     x12     y12
## KZ-0041 0.04815 0.04019 0.007034 0.007755 0.06873 -0.04266 0.08078 -0.1048
## KZ-0042 0.05221 0.04176 0.011682 0.009496 0.06649 -0.04276 0.07897 -0.1057
##            x13     y13    x14    y14    x15     y15    x16      y16    x17
## KZ-0041 0.1051 -0.1565 0.1046 0.1182 0.1716 0.05991 0.2955 0.002670 0.3516
## KZ-0042 0.0996 -0.1546 0.1059 0.1170 0.1737 0.06049 0.2979 0.003016 0.3524
##               y17    x18      y18     x19     y19     group
## KZ-0041 -0.002806 0.3641 -0.05098 -0.4852 0.04637 local bee
## KZ-0042 -0.001117 0.3677 -0.04858 -0.4793 0.04619 local bee
```

```
# Convert 2D array into a 3D array
wings.coords <- arrayspecs(KZ.lin[xyNames], p, k)
dimnames(wings.coords)[[3]] <- rownames(KZ.lin)

# Align the coordinates using Generalized Procrustes Analysis 
GPA.KZ.lin <- gpagen(wings.coords, print.progress = FALSE)

# Convert 3D array into a 2D array - opposite to arrayspecs
KZ.lin.aligned <- two.d.array(GPA.KZ.lin$coords)

# use equal prior probability for all groups
n.gr <- length(unique(KZ.lin$group)) # number of groups
KZ.lin.cva <- CVA(KZ.lin.aligned, KZ.lin$group, rounds = 10000, cv = TRUE,
                      prior = rep(1/n.gr, n.gr))
```

```
## singular Covariance matrix: General inverse is used. Threshold for zero eigenvalue is 1e-10
```

```
# Mahalanobis distances between groups
knitr::kable(as.data.frame(as.matrix(KZ.lin.cva$Dist$GroupdistMaha)))
```

|  | A | C | local bee | M | O |
| --- | --- | --- | --- | --- | --- |
| A | 0.000 | 8.513 | 7.960 | 8.061 | 6.091 |
| C | 8.513 | 0.000 | 7.562 | 11.155 | 8.008 |
| local bee | 7.960 | 7.562 | 0.000 | 11.886 | 7.207 |
| M | 8.061 | 11.155 | 11.886 | 0.000 | 10.209 |
| O | 6.091 | 8.008 | 7.207 | 10.209 | 0.000 |

```
# Significance of differences between groups
knitr::kable(as.data.frame(as.matrix(KZ.lin.cva$Dist$probsMaha)))
```

|  | A | C | local bee | M | O |
| --- | --- | --- | --- | --- | --- |
| A | 0e+00 | 1e-04 | 1e-04 | 1e-04 | 1e-04 |
| C | 1e-04 | 0e+00 | 1e-04 | 1e-04 | 1e-04 |
| local bee | 1e-04 | 1e-04 | 0e+00 | 1e-04 | 1e-04 |
| M | 1e-04 | 1e-04 | 1e-04 | 0e+00 | 1e-04 |
| O | 1e-04 | 1e-04 | 1e-04 | 1e-04 | 0e+00 |

```
KZ.lin.scores <- as.data.frame(KZ.lin.cva$CVscores)
# remove unwanted spaces in variable names otherwise use `CV 1`
colnames(KZ.lin.scores) <- gsub(" ", "", colnames(KZ.lin.scores)) 
KZ.lin.scores$group <- KZ.lin$group
rownames(KZ.lin.scores) = rownames(KZ.lin)

ggplot(KZ.lin.scores, aes(x = CV1, y = CV2, shape = group, color = group)) +
  geom_point() +
  scale_shape_manual(name ="", values = c(15, 18, 16, 17, 3),
                     breaks=c('local bee', 'A', 'C', 'M', 'O'),
                     labels=c('local bees', 'A', 'C', 'M', 'O')) +
  scale_color_manual(name ="", 
                     values = c("red", "orange", "blue", "green", "purple"),
                     breaks=c('local bee', 'A', 'C', 'M', 'O'),
                     labels=c('local bees', 'A', 'C', 'M', 'O')) +

  stat_ellipse()
```

```
CVA.class <- typprobClass(KZ.lin.cva$CVscores, groups = as.factor(KZ.lin$group), outlier = 0)
print(CVA.class)
```

```
##  cross-validated classification results in frequencies
##            
##              A  C local bee  M  O
##   A         85  0         0  0  0
##   C          0 37         0  0  0
##   local bee  0  0        29  0  0
##   M          0  0         0 16  0
##   O          1  0         0  0 48
## 
## 
##  cross-validated classification result in %
##            
##                    A        C local bee        M        O
##   A         100.0000   0.0000    0.0000   0.0000   0.0000
##   C           0.0000 100.0000    0.0000   0.0000   0.0000
##   local bee   0.0000   0.0000  100.0000   0.0000   0.0000
##   M           0.0000   0.0000    0.0000 100.0000   0.0000
##   O           2.0408   0.0000    0.0000   0.0000  97.9592
## 
## 
##  overall classification accuracy: 99.53704 %
## 
##  Kappa statistic: 0.99374
```

### Discrimination between local bees and subspecies from lineage O

```
KZ.sub = subset(sample.aligned, group == "local bee")

lin.tmp = sample.lin.aligned[, xy.Names]
rownames(lin.tmp) = sample.lin.aligned$sample
colnames(lin.tmp) = xyNames
lin.tmp$group = substr(rownames(lin.tmp),1,5)
lin.tmp = subset(lin.tmp, sample.lin.aligned$lineage == "O")

KZ.sub = rbind(KZ.sub, lin.tmp)
head(KZ.sub, 2)
```

```
##              x1       y1      x2       y2      x3      y3      x4       y4
## KZ-0041 -0.2784 -0.05707 -0.2361 -0.05729 -0.1949 0.06576 -0.1752 -0.01745
## KZ-0042 -0.2754 -0.05717 -0.2422 -0.05842 -0.1909 0.06448 -0.1810 -0.02003
##              x5      y5       x6      y6       x7     y7       x8      y8
## KZ-0041 -0.1563 -0.1487 -0.06587 0.07741 0.010014 0.1285 -0.01531 0.09147
## KZ-0042 -0.1611 -0.1465 -0.06797 0.07672 0.008286 0.1247 -0.01707 0.09101
##              x9      y9      x10      y10     x11      y11     x12     y12
## KZ-0041 0.04815 0.04019 0.007034 0.007755 0.06873 -0.04266 0.08078 -0.1048
## KZ-0042 0.05221 0.04176 0.011682 0.009496 0.06649 -0.04276 0.07897 -0.1057
##            x13     y13    x14    y14    x15     y15    x16      y16    x17
## KZ-0041 0.1051 -0.1565 0.1046 0.1182 0.1716 0.05991 0.2955 0.002670 0.3516
## KZ-0042 0.0996 -0.1546 0.1059 0.1170 0.1737 0.06049 0.2979 0.003016 0.3524
##               y17    x18      y18     x19     y19     group
## KZ-0041 -0.002806 0.3641 -0.05098 -0.4852 0.04637 local bee
## KZ-0042 -0.001117 0.3677 -0.04858 -0.4793 0.04619 local bee
```

```
#GPA re-alignment
# Convert 2D array into a 3D array
wings.coords <- arrayspecs(KZ.sub[xyNames], p, k)
dimnames(wings.coords)[[3]] <- rownames(KZ.sub)

# Align the coordinates using Generalized Procrustes Analysis 
GPA.KZ.sub <- gpagen(wings.coords, print.progress = FALSE)

# Convert 3D array into a 2D array - opposite to arrayspecs
KZ.sub.aligned <- two.d.array(GPA.KZ.sub$coords)

# use equal prior probability for all groups
n.gr <- length(unique(KZ.sub$group)) # number of groups
KZ.sub.cva <- CVA(KZ.sub.aligned, KZ.sub$group, rounds = 10000, cv = TRUE,
                      prior = rep(1/n.gr, n.gr))
```

```
## singular Covariance matrix: General inverse is used. Threshold for zero eigenvalue is 1e-10
```

```
# Mahalanobis distances between groups
knitr::kable(as.data.frame(as.matrix(KZ.sub.cva$Dist$GroupdistMaha)))
```

|  | local bee | O-adm | O-ana | O-arm | O-cau | O-cyp | O-med | O-syr |
| --- | --- | --- | --- | --- | --- | --- | --- | --- |
| local bee | 0.00 | 16.022 | 13.082 | 14.600 | 13.514 | 13.737 | 14.053 | 14.839 |
| O-adm | 16.02 | 0.000 | 8.857 | 10.209 | 13.462 | 9.679 | 9.860 | 11.197 |
| O-ana | 13.08 | 8.857 | 0.000 | 7.671 | 8.303 | 8.757 | 11.542 | 9.460 |
| O-arm | 14.60 | 10.209 | 7.671 | 0.000 | 9.978 | 7.654 | 9.412 | 10.382 |
| O-cau | 13.51 | 13.462 | 8.303 | 9.978 | 0.000 | 13.674 | 14.769 | 12.357 |
| O-cyp | 13.74 | 9.679 | 8.757 | 7.654 | 13.674 | 0.000 | 7.580 | 8.340 |
| O-med | 14.05 | 9.860 | 11.542 | 9.412 | 14.769 | 7.580 | 0.000 | 9.537 |
| O-syr | 14.84 | 11.197 | 9.460 | 10.382 | 12.357 | 8.340 | 9.537 | 0.000 |

```
# Significance of differences between groups
knitr::kable(as.data.frame(as.matrix(KZ.sub.cva$Dist$probsMaha)))
```

|  | local bee | O-adm | O-ana | O-arm | O-cau | O-cyp | O-med | O-syr |
| --- | --- | --- | --- | --- | --- | --- | --- | --- |
| local bee | 0e+00 | 0.0001 | 0.0001 | 0.0001 | 0.0001 | 0.0001 | 0.0001 | 0.0001 |
| O-adm | 1e-04 | 0.0000 | 0.0651 | 0.0139 | 0.0001 | 0.0517 | 0.0099 | 0.0015 |
| O-ana | 1e-04 | 0.0651 | 0.0000 | 0.1248 | 0.0304 | 0.1010 | 0.0013 | 0.0144 |
| O-arm | 1e-04 | 0.0139 | 0.1248 | 0.0000 | 0.0018 | 0.1809 | 0.0108 | 0.0029 |
| O-cau | 1e-04 | 0.0001 | 0.0304 | 0.0018 | 0.0000 | 0.0001 | 0.0001 | 0.0001 |
| O-cyp | 1e-04 | 0.0517 | 0.1010 | 0.1809 | 0.0001 | 0.0000 | 0.1427 | 0.0677 |
| O-med | 1e-04 | 0.0099 | 0.0013 | 0.0108 | 0.0001 | 0.1427 | 0.0000 | 0.0032 |
| O-syr | 1e-04 | 0.0015 | 0.0144 | 0.0029 | 0.0001 | 0.0677 | 0.0032 | 0.0000 |

```
KZ.sub.scores <- as.data.frame(KZ.sub.cva$CVscores)
# remove unwanted spaces in variable names otherwise use `CV 1`
colnames(KZ.sub.scores) <- gsub(" ", "", colnames(KZ.sub.scores)) 
KZ.sub.scores$group <- KZ.sub$group
rownames(KZ.sub.scores) = rownames(KZ.sub)

ggplot(KZ.sub.scores, aes(x = CV1, y = CV2, shape = group, color = group)) +
  geom_point() +
  scale_shape_manual(name ="", values = c(15, 18, 16, 17, 0:3),
                     breaks=c('local bee', 'O-adm', 'O-ana', 
                              'O-arm', 'O-cau',
                              'O-cyp', 'O-med', 'O-syr'),
                     labels=c('local bees', 'A. m. adami', 'A. m. anatolica', 
                              'A. m. armeniaca', 'A. m. caucasia', 
                              'A. m. cypria', 'A. m. meda', 'A. m. syriaca')) +
  scale_color_manual(name ="",
                     values = c("red", "orange", "blue", "green", 
                                     "purple", "black", "magenta", "gray70"),
                     breaks=c('local bee', 'O-adm', 'O-ana', 
                              'O-arm', 'O-cau',
                              'O-cyp', 'O-med', 'O-syr'),
                     labels=c('local bees', 'A. m. adami', 'A. m. anatolica', 
                              'A. m. armeniaca', 'A. m. caucasia', 
                              'A. m. cypria', 'A. m. meda', 'A. m. syriaca')) +

  stat_ellipse()
```

```
CVA.class <- typprobClass(KZ.sub.cva$CVscores, groups = as.factor(KZ.sub$group), outlier = 0)
print(CVA.class)
```

```
##  cross-validated classification results in frequencies
##            
##             local bee O-adm O-ana O-arm O-cau O-cyp O-med O-syr
##   local bee        29     0     0     0     0     0     0     0
##   O-adm             0     5     0     0     0     0     0     0
##   O-ana             0     0     5     0     0     0     0     0
##   O-arm             0     0     0     6     0     0     0     0
##   O-cau             0     0     0     0    12     0     0     0
##   O-cyp             0     0     0     0     0     4     0     0
##   O-med             0     0     0     0     0     0     8     0
##   O-syr             0     0     0     0     0     0     0     9
## 
## 
##  cross-validated classification result in %
##            
##             local bee O-adm O-ana O-arm O-cau O-cyp O-med O-syr
##   local bee       100     0     0     0     0     0     0     0
##   O-adm             0   100     0     0     0     0     0     0
##   O-ana             0     0   100     0     0     0     0     0
##   O-arm             0     0     0   100     0     0     0     0
##   O-cau             0     0     0     0   100     0     0     0
##   O-cyp             0     0     0     0     0   100     0     0
##   O-med             0     0     0     0     0     0   100     0
##   O-syr             0     0     0     0     0     0     0   100
## 
## 
##  overall classification accuracy: 100 %
## 
##  Kappa statistic: 1
```

### References

Nawrocka, A., Kandemir, İ., Fuchs, S., & Tofilski, A. (2018).
Computer software for identification of honey bee subspecies and
evolutionary lineages. Apidologie, 49(2), 172-184. https://doi.org/10.1007/s13592-017-0538-y

Nawrocka, A., Kandemir, İ., Fuchs, S., & Tofiilski, A. (2018).
Dataset: Computer software for identification of honey bee subspecies
and evolutionary lineages. Apidologie, 49, 172–184. https://doi.org/10.5281/zenodo.7567336

### Information about session

```
sessionInfo()
```

```
## R version 4.0.3 (2020-10-10)
## Platform: x86_64-w64-mingw32/x64 (64-bit)
## Running under: Windows 10 x64 (build 19043)
## 
## Matrix products: default
## 
## locale:
## [1] LC_COLLATE=Polish_Poland.1250  LC_CTYPE=Polish_Poland.1250   
## [3] LC_MONETARY=Polish_Poland.1250 LC_NUMERIC=C                  
## [5] LC_TIME=Polish_Poland.1250    
## 
## attached base packages:
## [1] stats     graphics  grDevices utils     datasets  methods   base     
## 
## other attached packages:
##  [1] ggspatial_1.1.6     raster_3.5-21       sp_1.5-0           
##  [4] rnaturalearth_0.1.0 ggrepel_0.9.1       ggforce_0.3.3      
##  [7] ggplot2_3.3.6       MASS_7.3-58.1       Morpho_2.9         
## [10] shapes_1.2.6        geomorph_4.0.4      Matrix_1.4-1       
## [13] rgl_0.109.6         RRPP_1.3.0         
## 
## loaded via a namespace (and not attached):
##  [1] sass_0.4.1              jsonlite_1.8.0          foreach_1.5.2          
##  [4] bslib_0.4.1             assertthat_0.2.1        highr_0.9              
##  [7] yaml_2.3.5              pillar_1.8.1            lattice_0.20-41        
## [10] glue_1.6.2              digest_0.6.29           polyclip_1.10-0        
## [13] colorspace_2.0-3        htmltools_0.5.3         pkgconfig_2.0.3        
## [16] s2_1.1.2                Rvcg_0.21               scales_1.2.1           
## [19] tweenr_1.0.2            terra_1.6-7             jpeg_0.1-9             
## [22] tibble_3.1.8            proxy_0.4-27            generics_0.1.3         
## [25] farver_2.1.1            cachem_1.0.6            withr_2.5.0            
## [28] cli_3.3.0               magrittr_2.0.1          evaluate_0.18          
## [31] fansi_1.0.3             doParallel_1.0.17       nlme_3.1-149           
## [34] class_7.3-17            tools_4.0.3             minpack.lm_1.2-2       
## [37] formatR_1.14            lifecycle_1.0.1         stringr_1.4.0          
## [40] munsell_0.5.0           colorRamps_2.3.1        bezier_1.1.2           
## [43] compiler_4.0.3          jquerylib_0.1.4         e1071_1.7-11           
## [46] rlang_1.0.4             classInt_0.4-7          units_0.8-0            
## [49] grid_4.0.3              iterators_1.0.14        rstudioapi_0.14        
## [52] htmlwidgets_1.5.4       labeling_0.4.2          base64enc_0.1-3        
## [55] rmarkdown_2.18          wk_0.6.0                gtable_0.3.1           
## [58] codetools_0.2-16        DBI_1.1.3               R6_2.5.1               
## [61] rgdal_1.5-32            knitr_1.40              dplyr_1.0.9            
## [64] fastmap_1.1.0           utf8_1.2.2              KernSmooth_2.23-17     
## [67] ape_5.4-1               stringi_1.7.8           parallel_4.0.3         
## [70] Rcpp_1.0.9              vctrs_0.4.1             sf_1.0-7               
## [73] rnaturalearthdata_0.1.0 scatterplot3d_0.3-42    tidyselect_1.2.0       
## [76] xfun_0.31
```
