# Supplementary figures and images for: In Search of Apis mellifera pomonella in Kazakhstan
Source: Life (Basel). 2023 Sep 3;13(9):1860. doi: 10.3390/life13091860 (PMC10532534; doi:10.3390/life13091860)

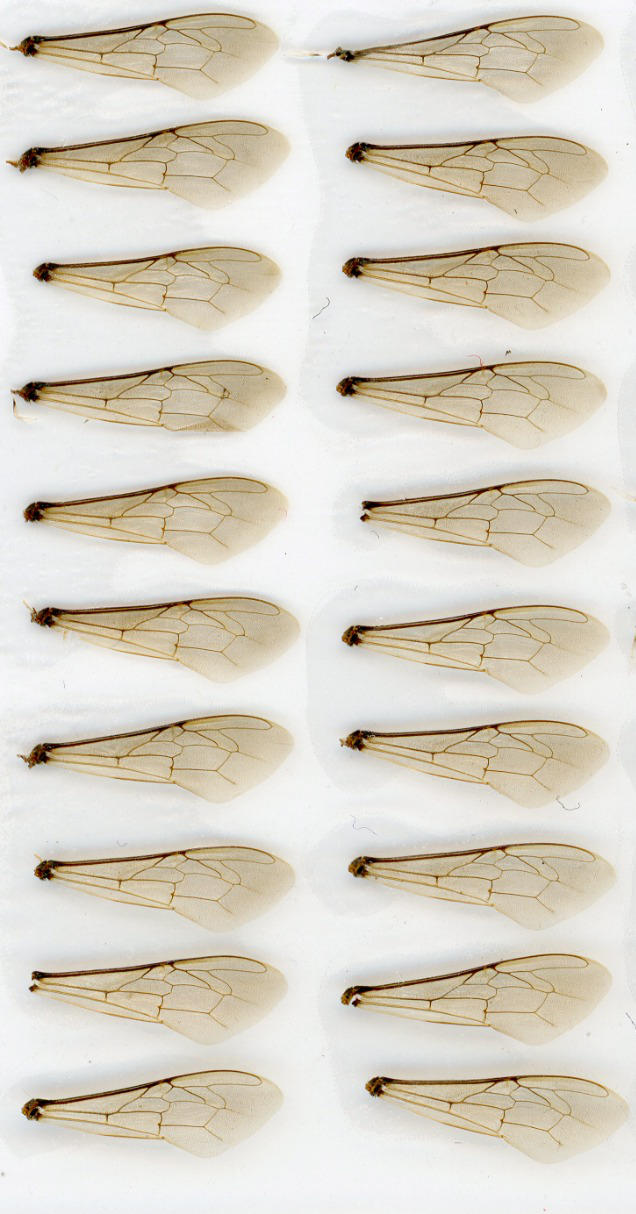

Supplement: Supplementary file 1 [file life-13-01860-s001.zip › Supplementary-figure-S1.jpeg]
